# Targeted Antenatal Screening for Predicting Postpartum Thyroiditis and Its Evolution Into Permanent Hypothyroidism

**DOI:** 10.3389/fendo.2020.00220

**Published:** 2020-04-17

**Authors:** Salvatore Benvenga

**Affiliations:** ^1^Department of Clinical and Experimental Medicine, University of Messina, Messina, Italy; ^2^Master Program on Childhood, Adolescent and Women's Endocrine Health, University of Messina, Messina, Italy; ^3^Interdepartmental Program on Molecular and Clinical Endocrinology, and Women's Endocrine Health, University Hospital, A.O.U. Policlinico G. Martino, Messina, Italy

**Keywords:** thyroid autoimmunity, pregnancy, postpartum, fish consumption, autoimmune diseases, autoimmune thyroid diseases, postpartum thyroiditis, antenatal screening

## Abstract

Postpartum thyroiditis (PPT) has a prevalence of 1–22%, with an ~50% rate of evolution into permanent hypothyroidism (PH). PPT risk is assessed by measuring serum thyroid antibodies during gestation, as 1/3–1/2 of Ab+ve pregnant women will develop PPT. Family and personal history positive for autoimmune non-thyroid diseases (AINTDT), and consumption of swordfish increases while consumption of small oily fish decreases the risk of PPT. Monitoring thyroid function in a very high-risk subgroup avoids the costs of the Ab-based universal screening. We aimed at identifying such subgroup in 412 women followed from week 7–11 of gestation to month 12 postpartum. At study entry, we measured serum TPOAb, TgAb, TSH, FT4, FT3, and evaluated seafood consumption, familial history for thyroid diseases and AINTD, and personal history for AINTD. We measured TSH, FT4, FT3 at 1.5, 3, 6, and 12 months postpartum. PPT occurred in 63 women (15.3%), and PH in 34/63 (54%). Based on positivity/negativity for the three histories, women were classified into 8 categories, with PPT rates of 3.8–100%. Seafood consumption allowed further separation of subgroups having different PPT risks. We considered 11 possible strategies, termed [a] through [k]. Strategy [a] consisted in omitting gestational screening, while performing universal postpartum monitoring with TSH and one thyroid hormone; strategy [k] consisted in selective gestational screening with TPOAb and TgAb, based on history and fish consumption, and selective postpartum monitoring in TPOAb and/or TgAb+ve women. The 100% sensitivity, specificity and diagnostic accuracy of strategy [a] were counterbalanced by the highest costs (Euro 32,960 or 523 per each PPT caught). The corresponding numbers for strategy [k] were 78, 95, 93%, and Euro 8,920 or 182/PPT caught. These savings stem from gestational screening being done in 186 women, and postpartum monitoring done in 65/186 women. One gestational screning-free strategy was the cheapest (Euro 2,080 or 83/PPT caught), because based on postpartum monitoring of only 26 women, but had the lowest sensitivity (40%). Identification of pregnant women having different risks for PPT is feasible, with the costless evaluation of history and seafood consumption driving gestational screening of thyroid antibody status and postpartum monitoring of thyroid function.

## Introduction

Postpartum thyroiditis (PPT) is a destructive autoimmune inflammation in women who did not have overt thyroid disease before pregnancy, and it has a prevalence of 1–22% depending on geographic area (1, 2). Thyroid dysfunction can be biphasic (thyrotoxicosis followed by hypothyroidism) or monophasic (thyrotoxicosis only or hypothyroidism only), with an ~50% rate of no return to euthyroidism at the end of the 12th month postpartum, a condition termed permanent hypothyroidism (PH). (1, 2) If not evolved into PH, PPT tends to recur (1).

Women at risk for PPT are classically identified by screening with measurement of serum thyroid autoantibodies (TPOAb or, less commonly, both TPOAb and TgAb) during the first trimester of gestation, as one-third to one-half of thyroid Ab+ve pregnant women will develop PPT (1). Personal history of autoimmune non-thyroid disease (AINTD; e.g., type 1 diabetes mellitus [DM-1], lupus erythematosus systemicus), family history of thyroid disease, (1–3) and familial history of AINTD, (2) but not personal history of miscarriages or smoking status (1, 2) are risk factors for PPT. Concerning the understimated favoring role for development of PPT given by familial history of AINTD, in our study on a cohort of 412 women who were followed-up from week 7–11 of gestation through the end of the 12th month postpartum, (2) we found that the magnitude of risk conferred by familial history of AINTD (43/63 [68.2%] in the PPT group vs. 148/349 [42.4%] in the non-PPT group, *P* = 0.0002, OR = 2.92) was comparable to that conferred by personal history of AINTD (29/63[46.0%] vs. 80/349 [22.9%], *P* = 0.0001, OR = 2.87), and greater than that conferred by family history of thyroid disease (27/63 [42.9%] vs. 100/349 [28.7%], *P* = 0.025, OR = 1.87) (2).

Recently, we postulated (4) and then verified (5) that consumption of no fish (that is, meat eating) increases and, among fish eaters, swordfish consumption also increases the risk of PPT, while consumption of small oily fish decreases the risk of PPT. In that study, (5) which was based on universal screening by TPO and TgAb, the prevalence of PPT was 15.3%. This is the second highest frequency of PPT in Italy after the 22.1% found in Liguria, (6) this last prevalence matching the 22.3% of Wales (7).

There is no consensus on screening for PPT (1). Both the 2011 (8) and 2017 (9) guidelines of the American Thyroid Association (ATA) limit the postpartum search for PPT in women with postpartum depression (Recommendation 63 “*Women with postpartum depression should have TSH, FT4, and TPOAb tests performed. Level B-USPSTF*”; (8) Recommendation 85 “*All patients with depression, including postpartum depression, should be screened for thyroid dysfunction. Strong recommendation, low-quality evidence*” (9)) According to the 2017 ATA guidelines, (9) universal screening for TPOAb in early pregnancy or possibly preconception is attractive, but it warrants further investigation. In an approach-to-the patient article, (1). Stagnaro-Green underscores that “*identifying women at risk for developing PPT could result in focused screening in the postpartum. TPO-Ab measured in the first trimester is the optimal screening tool* […]. *The present author also recommends that women who have had a prior history of PPT, who have another autoimmune disease, or are known to be TPO-Ab*+ *be screened at 3 months for PPT with a TSH and reflex FT4. If they are euthyroid and TPO-Ab*+*, no further screening is indicated. However, if they are TPO-Ab- and euthyroid, TSH levels should be obtained at 6 and 9 months after delivery*” (1).

We reasoned that upon simply combining the information on the fish consumption with both the said personal and family histories, and without performing gestational universal screening, we could have predicted a significant proportion of women at very high risk for developing PPT on one side and a significant proportion of women at very minimal risk on the other side. If so, one could perform a targeted biochemical gestational screening, by sparing it to these two categories of women and reserving it to the remaining women. A secondary interest was to see whether we could have used the same combination of information to even predict evolution of PPT into PH. Finally, we wished to compare the strategy of targeted screening (using both TPOAb and TgAb, either one considered positive when above the midpoint of the reference range) with the strategy of universal screening using the classical marker at the conventional threshold (serum TPOAb, considered positive when above the upper limit of the reference range).

## Materials and Methods

The cohort of the 412 singleton pregnancy women and with no known thyroid disease, whose enrolment occurred at 7–11 weeks of gestation, was described in detail previously (2, 5). Particularly, a questionnaire was administered to investigate on family and personal history for TD, and AINTD as well. If necessary, and upon consent, clinical records of either the pregnant woman or consanguineous relatives were examined. Age at enrollment was 31.6 ± 4.3 years (range 19–43). Of the 412 women, approximately three-fourths were parous, one-seventh had a previous miscarriage, and one-fourth either current or previous smokers.

Concerning fish consumption, rationale and details for creating fish groups were also provided previously (4). For purposes of consistency with our previous papers (4, 5) fish groups were termed A through E. Thus, group A (*n* = 92) consists of women with selective or predominant consumption of swordfish. Group B (*n* = 85) consists of women with selective or predominant consumption of oily fish. Group C (*n* = 108) consists of women who consume swordfish plus other fish, with swordfish consumption occurring infrequently; if eaten, oily fish also was consumed infrequently. Group D (*n* = 117) consists of women who consume fish other than swordfish and oily fish. Group E (*n* = 10) consists of women who did not consume fish at all (meat eaters). The terms “predominant” or “infrequent” indicate at least 50% of the total monthly fish consumprion or <50% of the total monthly fish consumption (4), respectively.

The study, in the context of a program for the Health Service Development of Sicily, was conducted in accordance with the ethical standards of our institutional research Committee, the 1964 Declaration of Helsinki, and its later amendments. Informed consent was obtained from all the participants.

### Costs of Screening

We calculated the costs of gestational screening of women at risk for PPT, if performed, and the costs of postpartum screening (that we prefer to call postpartum monitoring) of thyroid function in women considered to be at PPT risk based on gestational screening. We prefer the terminology of postpartum monitoring because thyroid dysfunction occurs anytime between the first weeks after delivery and the 12th month of gestation; furthermore, measurement of thyroid function tests at the end of the first year postpartum tells us if euthyroidism is restored or hypothyroidism persists. Because of minimal differences among the costs of TSH, FT4, FT3, TPOAb, and TgAb, at least in our geographical area, the out-of-pocket cost for any of these five analytes was rounded off to Euro 10.

Costs of gestational screening were based on assay of TPOAb (Euro 10 for one determination in one woman) or assay of both TPOAb and TgAb (Euro 20 for one determination of the two Ab in one woman). Costs of postpartum monitoring of thyroid function were based on assay of TSH and FT4 at four time points during the first 12 months postpartum. Such frequency of biochemical assay is compatible with thyrotoxicosis occurring most frequently between months 2 and 6, and hypothyroidism between months 3 and 12. Opting for FT3, in lieu of FT4, is inconsequential, because costs for FT4 and FT3 coincide. Thus, postpartum measurement of TSH and one thyroid hormone four times during the first 12 months postpartum equates to Euro 80 per each woman.

### Statistics

Comparisons between proportions of categorical variables were performed using the χ^2^ test or Fisher's exact test, as appropriate. The Bonferroni test was applied to account for multiple comparisons. The level of statistical significance was always set at *P* <0.05. *P*-values between 0.10 and 0.05 were considered borderline significant.

## Results

Before analyzing the value of combining the three types of history with the pattern of fish consumption, it is of interest to analyze the value of combining the three types of history alone.

### Personal History of AINTD, Family History of AINTD, Family History of TD

There are 8 possible combinations (indicated as I through VIII in Table 1), corresponding to 8 categories of women. Table 1 also summarizes the level of statistical difference for frequencies of both PPT and PH in each category as compared with each of the other 7 categories. It is evident that the common denominator between the three categories with the top frequency of PPT (≥50%; categories II, III and IV) is personal history positive for AINTD.

**Table 1:** Prevalence of postpartum thyroiditis (PPT) and PPT evolution into permanent hypothyroidism (PH) in 412 pregnant women who were stratified into 8 categories (I through VIII) based on posivity or negativity for each of three histories.

	**Categories**							
	**I**	**II**	**III**	**IV**	**V**	**VI**	**VII**	**VIII**
Familial history of TD	–	+	+	–	–	+	–	+
Familial history of AINTD	–	+	–	+	–	–	+	+
Personal history of AINTD	–	+	+	+	+	–	–	–
Prevalence of category in our cohort (*n* = 412)	93 (22.6%)	12 (2.9%)	4 (1%)	15 (3.6%)	78 (18.9%)	46 (11.2%)	103 (25%)	61 (14.8%)
Prevalence of PPT (*n* = 63) in the category	9/93 [9.7%]	12/12 [100%]	2/4 [50%]	12/15 [80%]	3/78 [3.8%]	6/ 46 [14.5%]	12/103 [11.6%]	7/61 [11.5%]
Statistics (difference in % of PPT between categories)								
vs I	N/A	χ^2^ =54.2		χ^2^ = 40.8				
		***P*** **= 2.0 × 10**^**−11**^	*P* = 1.0	***P*** **= 4.8 × 10**^**−9**^	*P* = 1.0	*P* = 1.0	*P* = 1.0	*P* = 1.0
vs II		N/A			χ^2^ = 69.2	χ^2^ = 33.6	χ^2^ = 50.8	χ^2^ = 40.8
			*P* = 1.0	*P* = 1.0	***P*** **= 1.7 × 10**^**−14**^	***P*** **= 5.3 × 10**^**−7**^	***P*** **= 9.8 × 10**^**−11**^	***P*** **= 1.4 × 10**^**−8**^
vs III			N/A	*P* = 1.0	*P* = 0.48	*P* = 1.0	*P* = 1.0	*P* = 1.0
vs IV				N/A	χ^2^ = 53.9	χ^2^ = 24.4	χ^2^ = 37.7	χ^2^ = 30.1
					***P*** **= 5.6 × 10**^**−12**^	***P*** **= 2.2 × 10**^**−5**^	***P*** **= 2.2 × 10**^**−8**^	***P*** **= 1 × 10**^**−6**^
vs V					N/A			
						*P* = 1.0	*P* = 1.0	*P* = 1.0
vs VI						N/A		
							*P* = 1.0	*P* = 1.0
vs VII							N/A	
								*P* = 1.0
vs VIII								N/A
Prevalence of PH (*n* = 34) in the PPT cases of the category	4/9 (44.4%)	10/12 (83.3%)	1/2 (50%)	5/12 (41.7%)	1/3 (33.3%)	1/6 (16.7%)	6/12 (50%)	6/7 (85.7%)

*AINTD, autoimmune non-thyroid disease; PH, permanent hypothyroidism; PPT, postpartum thyroiditis; TD, thyroid disease*.

*P-values typed boldface indicates statistical significance (P < 0.05 minimum). P-values were corrected for multiple comparisons using the Bonferroni's test*.

*Concerning statistics for frequencies of PH (based on Fisher's exact test with Bonferron's correction for multiple comparisons), no group differed from any of the other 7 groups*.

The first two combinations are the extreme ones (category I = all three histories negative; category II = all three histories positive). In category I, there were 93/412 women (22.6%), of whom only 9 (9.7%) went on to develop PPT. Based on this low risk, one could opt to spare the biochemical screening for PPT and, accordingly, to spare follow-up in the first year after delivery (for detecting thyroid dysfunction) to approximately one-fifth of pregnant women. By doing so in these 93 category I women, one misses 9 of a total 63 women (14.3%) who will develop PPT. One also misses 4 of a total of 34 women (11.8%) with PH. By contrast, in category II, which consists of 12/412 women (2.9%), all 12 (100%) went on to develop PPT, accounting for 12/63 (19%) of all PPT women; 10/12 (83.3%) PPT progressed to PH. Hence, one could opt to spare the biochemical screening also to another 3% of women, as this 3% is destined to develop PPT, bringing the total of gestational screening-free women to 105/412 (25.5%). However, unlike category I, category II women will be monitored closely in the postpartum. In brief, assuming that these two categories have the same frequency as in our cohort, once they are identified by simply taking their histories, money will be saved by omitting gestational screening to one-fourth of the pregnant women in the cohort and by performing postpartum monitoring of thyroid function in 3% of the women in the cohort.

There were another two interesting categories, due to their extreme rates of PPT. In category V, which accounted for 78/412 (18.9%) of women and in whom solely personal history for AINTD was positive, only 3 (3.8%) developed PPT (Table 1). In contrast, in category IV, which accounted for 15/412 women (3.6%), and in whom both personal and familial histories for AINTD were positive, 12 (80%) developed PPT. Pooling these data of categories IV and V with those for categories I and II, one could exclude from gestational screening a total of 198/412 women (48.1%). By doing so, one would miss a total of 12/63 women (19%; 9 in category I and 3 in category V) who will develop PPT, but would capture 24/63 women who will develop it (38.1%; 12 in category II and 12 in category IV) upon monitoring postpartum thyoid function in the 12 women of category II and 15 women of category IV. In brief, one can spare the gestational biochemical screening to virtually half of the pregnant women (198/412; namely 93 of category I, 12 of category II, 15 of category IV and 78 of category V) and correctly catch almost 40% of all PPT women (and 44% of the PH women [15/34]) while missing 20% of them (and 15% of all PH women [5/34]) (Table 1).

If one adds category III (*n* = 4/412 [0.97%], with 2/4 developing PPT and 0/2 progressing to PH), in which only familial history for AINTD is negative, then gestational screening is spared to 202/412 women (49%); 26/63 women (41.3%) will be correctly considered as destined to develop PPT, and 14/63 (22.2%) who are destined to develop PPT will be missed. Gestational screening is spared, but postpartum monitoring of thyroid function is performed in half of the cohort (222/412, namely 12 of category II, 4 of category III,15 of category IV plus 210 of categories VI–VIII).

### Combining Type of Seafood Consumption With any of Personal History Positive for AINTD, Family History Positive for AINTD, and Family History Positive for TD

Applying the additional information of fish consumption to the aforementioned five categories (I, II, III, IV, and V) is rewarding in terms of minimizing the number of women destined to develop PPT who are screened only verbally (i.e., taking the three histories and omitting inquiry about fish consumption) (Table 2). Of the 9 PPT women missed in category I upon omitting both gestational screening and postpartum monitoring in all category I women, 2 (22.2%) were in group A (swordfish eaters), accounting for 2/9 (22.2%) group A women in category I. Of the 2 PPT women missed in category III, both were in group A, accounting for 2/2 group A women in category III. Of the 3 PPT women missed in category V, 2 (66.7%) were in group A, accounting for 2/25 (8.0%) group A women in category V (Table 2). Thus, close attention to monitoring postpartum thyroid dysfunction starting from early after delivery has to be paid to group A women in categories II, III, and IV, because a total of 11/11 (5/5, 2/2 and 4/4, respectively) will go on to develop PPT, with evolution to PH in 6/11.

**Table 2:** Prevalence of postpartum thyroiditis (PPT) and PPT evolution into permanent hypothyroidism (PH) in 412 pregnant women who were stratified into 8 categories (I through VIII) based on posivity or negativity for each of three histories, and substratified based on fish consumption.

	**Categories**							
	**I**	**II**	**III**	**IV**	**V**	**VI**	**VII**	**VIII**
	**(*n* = 93)**	**(*n*= 12)**	**(*n* = 4)**	**(*n* = 15)**	**(*n* = 78)**	**(*n* = 4)**	**(*n* = 103)**	**(*n* = 61)**
Familial history of TD	–	+	+	–	–	+	–	+
Familial history of AINTD	–	+	–	+	–	–	+	+
Personal history of AINTD	–	+	+	+	+	–	–	–
Group A (*n* = 92)	9	5	2	4	25	9	23	15
PPT (*n* = 22)	2/9 (22%)	5/5 (100%)	2/2 (100%)	4/4 (100%)	2/25 (8%)	2/9 (22%)	2/23 (9%)	3/15 (20%)
PH (*n* = 12)	0/2	3/5 (60%)	1/2 (50%)	2/4 (50%)	1/2 (50%)	0/2	2/2 (100%)	3/3 (100%)
Group B (*n* = 85)	32	0	0	3	8	11	19	12
PPT (*n* = 4)	0/32	0	0	1/3 (33%)	1/8 (12%)	1/11 (9%)	0/19	1/12 (8%)
PH (*n* = 2)	0/0	0	0	0/1	0/1	1/1	0/0	1/1
Group C (*n* = 108)	25	3	2	3	20	10	27	18
PPT (*n* = 17)	2/25 (8%)	3/3 (100%)	0/2	3/3 (100%)	0/20	1/10 (10%)	7/27 (26%	1/18 (6%)
PH (*n* = 9)	2/2 (100%)	3/3 (100%)	0/0	1/3 (33%)	0/0	1/1	2/7 (29%)	1/1
Group D (*n* = 117)	26	3	0	3	24	14	31	16
PPT (*n* = 16)	5/26 (19%)	3/3 (100%)	0	2/3 (67%)	0/24	2/14 (14%)	2/31 (6%)	2/16 (12%)
PH (*n* = 9)	2/5	3/3 (100%)	0	1/2 (50%)	0/0	0/2	2/2 (100%)	1/2 (50%)
Group E (*n* = 10)	1	1	0	2	1	2	3	0
PPT (*n* = 14)	0/1	1/1	0	2/2 (100%)	0/1	0/2	1/3 (33%)	0
PH (*n* = 2)	0/0	1/1	0	1/2 (50%)	0/0	0/0	0/1	0
Total (*n* = 412)	93	12	4	15	78	46	103	61
PPT (*n* = 63)	9/93 (10%)	12/12 (100%)	2/4 (50%)	12/15 (80%)	3/78 (4%)	6/46 (13%)	12/103 (12%)	7/61 (11%)
PH (*n* = 34)	4/9 (44%)	10/12 (83%)	1/2 (50%)	5/12 (42%)	1/3 (33%)	1/6 (17%)	6/12 (50%)	6/7 (86%)

*AINTD, autoimmune non-thyroid disease; PH, permanent hypothyroidism; PPT, postpartum thyroiditis; TD, thyroid disease*.

*For definition of groups (A through E) based on fish consumption, see Materials and Methods. As reported previously (4, 5), the fish portion eaten by all women was invariably the 150 g one. Because seafood was eaten as an entrée either at lunch or dinner, for statistical purposes, number of fish portions consumed each month and frequency of fish consumed each month coincided. Confirming the 7.0–7.8 range of monthly fish consumption found in the previous study on 236 women (4), the four fish groups remained comparable in terms of frequency of fish consumption (7.5–8.0 times/month or twice a week) in the 412 women (5)*.

In contrast, such postpartum monitoring can be spared to group A women in categories I and V (the two categories that share negative history for both familial TD and familial AINTD), because the risk of PPT is 2/9 (22.2%) and 2/25 (8.0%), respectively, with an overall evolution to PH of 1/4 (25%). The risk of PPT remains below 25% also in group A women of categories VI, VII, and VIII (Table 2). Concerning group B women (who were absent in groups II and III), no gestational screening and no subsequent postpartum monitoring is needed for categories I and VII, because the PPT risk is 0/32 and 0/19, respectively. The risk of PPT for group B women is minimal in categories VIII, VI and V (1/12 [8.3%], 1/11 [9.1%], and 1/8 [12.5%]), but significant in category IV (1/3 [33.3%]) (Table 2). Concerning group C, D and E women, the PPT risk is very high in categories II and IV, but absent or minimal in the other categories (Table 2).

### Summary

Gestational screening can be omitted, but postpartum thyroid function monitoring cannot, in 26/412 (6.3%) women with extremely high propensity to develop PPT (25/26 [96%]) and high risk of progression to PH (16/25 [64%]). These women are all of those in category II, regardless of seafood consumption, group A women in category III, and all of those in category IV except group B. For the opposite reason, both gestational screening and postpartum thyroid function monitoring can be omitted in the following 200/412 (48.6%) women with scarce propensity to develop PPT (7/200 [3.5%]), though with high risk of PPT progression to PH (6/7 [85.7%]). These 200 women are category I (groups B, C, and E; PPT rate = 2/58 [3.4%]); category III (group C; PPT rate = 0/2); category V (groups A, C, D, and E; PPT rate= 2/70 [2.8%]); category VI (group E; PPT rate = 0/2); category VII (groups B and D; PPT rate= 2/50 [4%]); category VIII (group C; PPT rate = 1/18 [5.6%]). Hence, both gestational screening and postpartum thyroid function monitoring are performed in the remaining 186/412 women (45.1%), who have a moderate risk of developing PPT (31/186 [16.7%]) and PPT progression to PH (12/31 [38.7%]). These 186 women are in category I (groups A and D; PPT rate = 7/35 [20%]); category IV (group B; PPT rate= 1/3 [33.3%]); category V (group B; PPT rate = 1/8 [12.5%]); category VI (groups A, B, C, and D; PPT rate = 6/44 [13.6%]); category VII (groups A, C, and E; PPT rate = 10/53 [18.9%]); category VIII (groups A, B, and D; PPT rate = 6/43 [14.0%]).

### Costs of Selected Gestational Screening and Postpartum Monitoring vs. Other Strategies

Diagnostic performances of strategies (that we term [a] through [k]) and associated costs are summarized in Table 3. Costs are given for our cohort of 412 women and projected for a cohort of 1,000 women (last line in Table 3). The strategy of omitting any gestational biochemical screening in favor of universally measuring thyroid function (TSH and one thyroid hormone) at four time points in the first year postpartum is obviously the most rewarding but also the most expensive strategy (Table 3, **first column, strategy [a]**). For a cohort like ours, this strategy translated into a total cost of almost Euro 33,000, equating to Euro 523 per each case of PPT detected or Euro 969 per each PH detected. The other strategies select the population of women who will undergo the postpartum biochemical check of thyroid function. With strategies [b] or [c], this selection is at no cost because based, respectively, on history taking or history taking plus questionnaire of fish consumption. As explained in the preceding headings, these strategies allow to restrict postpartum assays to about 7% of the initial cohort of pregnant women, resulting in the lowest cost in absolute terms (<Euro 5,000) and per each case of PPT detected (<Euro 100) or PH detected (<Euro 200) but also the lowest yield, with only 40% of PPT cases being detected.

Table 3Statistical characteristics and costs (in Euro) of different strategies to screen for postpartum thyroiditis (PPT).

**Table:** 

	**Strategies for gestational screening and postpartum monitoring**						
	**[a]**	**[b]**	**[c]**	**[d]**	**[e]**	**[f]**	**[g]**
**Gestational screening**	Not done	Universal	Universal	Universal	Universal	Universal	Universal
Histories	Not done	Yes	Yes	Not done	Not done	Not done	Not done
Fish consumption	Not done	Not done	Yes	Not done	Not done	Not done	Not done
Assay	Not done	Not done	Not done	TPOAb	TPOAb	TPOAb & TgAb	TPOAb & TgAb
Population assayed	0	0	0	412	412	412	412
Cost	0	**0**	**0**	4,120	4,120	8,240	8,240
**Postpartum monitoring**	Universal	Selective	Selective	Selective	Selective	Selective	Selective
Population monitored	412	31 +ve [categ II, III, IV]	26 +ve [categ II, all groups categ III, group A cat IV, groups A,C, D,E]	56 +ve (TPOAb >100 U/ml)	68 +ve (TPOAb >60 U/ml)	76 +ve (TPOAb and/or TgAb >100 U/ml)	93 +ve (TPOAb and/or TgAb >60 U/ml)
Sensitivity	63/63 (100%)	26/63 (41.3%)	25/63 (39.7%)	39/63 (61.9%)	42/63 (66.7%)	48/63 (76.2%)	54/63 (**85.7%**)
Specificity	349/349 (100%)	344/349 (98.6%)	348/349 (**99.7%**)	323/349 (95.1%)	323/349 (92.5%)	321/349 (92.0%)	310/349 (88.8%)
PPV	63/63 (100%)	26/31 (83.9%)	25/26 (**96.2%**)	39/56 (69.6%)	42/68 (61.8%)	48/76 (63.2%)	54/93 (58.1%)
NPV	349/349 (100%)	344/381 (90.3%)	348/386 (90.2%)	332/356 (93.3%)	323/344 (93.9%)	321/336 (95.5%)	310/319 (**97.2%**)
False +ve	0/63	5/31 (16.1%)	1/26 (**3.8%**)	17/56 (30.4%)	26/68 (38.2%)	28/76 (36.8%)	39/93 (41.9%)
False –ve	0/349	37/381 (17.6%)	38/386 (9.8%)	24/356 (6.7%)	21/344 (6.1%)	15/321 (4.7%)	9/319 (**2.8%**)
PPT missed	0	37/63 (58.7%)	38/63 (60.4%)	24/63 (38.1%)	21/63 (33.3%)	15/63 (23.8%)	9/63 (**14.3%**)
Diagnostic accuracy	412/412 (100%)	350/412 (84.9%)	373/412 (90.5%)	371/412 (90%)	365/412 (88.6%)	369/412 (89.6%)	364/412 (88.3%)
Cost	32,960	2,480	2,080	4,480	5,440	6,080	7,440
Total cost	32,960	2,480	**2,080**	8,600	9,560	14,320	15,680
Total cost per each PPT caught	523.20	95.40	**83.20**	220.51	227.62	298.33	290.37
Total cost per each PH caught	969.41	155	130	373.91	398.33	511.43	540.70
Total cost if cohort is 1,000 women instead of 412	80,000	6,000	**5,040**	20,880	23,200	34,800	38,100

**Table:** 

	**Strategies for gestational screening and postpartum monitoring**			
	**[h]**	**[i]**	**[j]**	**[k]**
**Gestational screening**	Selective	Selective	Selective	Selective
Histories	Yes	Yes	Yes	Yes
Fish consumption	Yes	Yes	Yes	Yes
Assay	TPOAb	TPOAb	TPOAb & TgAb	TPOAb & TgAb
Population assayed	186	186	186	186
Cost	1,860	1,860	3,720	3,720
**Postpartum monitoring**	Selective	Selective	Selective	Selective
Population monitored	45, namely 19/186 TPOAb+ve (>100 U/ml) from gestational screening and 26 from no screening	51, namely 25/186 TPOAb+ve (>60 U/ml) from gestational screening and 26 from no screening	58, namely 32/186 TPOAb and/or TgAb +ve (>100 U/ml) from gestational screening and 26 from no screening	65, namely 39/186 TPOAb and/or TgAb +ve (>60 U/ml) from gestational screening and 26 from no screening
Sensitivity, True +ve	40/63 (63.5%)	42/63 (66.7%)	46/63 (73.0%)	49/63 (77.8%)
Specificity, True -ve	344/349 (98.6%)	340/349 (97.4%)	337/349 (96.6%)	333/349 (95.4%)
PPV	40/4 5 (88.9%)	42/51 (82.4%)	46/58 (79.3%)	49/65 (75.4%)
NPV	344/367 (93.7%)	340/361 94.2%)	337/354 (95.2%)	333/347 (96.0%)
False +ve	5/45 (11.1%)	9/51 (17.6%)	12/58 (20.7%)	16/65 (24.6%)
False –ve	23/367 (6.3%)	21/361 (5.8%)	17/354 (4.8%)	14/347 (4.0%)
PPT missed	23/63 (36.5%)	21/63 (33.3%)	17/63 (27.0%)	14/63 (22.2%)
Diagnostic accuracy	384/412 (**93.2%**)	382/412 (92.7%)	383/412 (93.0%)	382/412 (92.7%)
Cost	3,600	4,080	4,640	5,200
Total cost	5,460	5,940	8,360	8,920
Total cost per each PPT caught	136.50	141.43	181.74	182.04
Total cost per each PH caught	248.18	270	334.40	356.80
Total cost if cohort is 1,000 women instead of 412	13,230	14,430	20,300	21,660

*NPV, Negative predictive value; PPV, Positive predictive value. Diagnostic accuracy is [True +ve plus True -ve] divided by all 412 women*.

*Costs are in Euro, with Euro 10/00 being the cost for the assay of any of TgAb, TPOA, TSH, thyroid hormone. Postpartum monitoring by measuring serum TSH and one free thyroid hormone (FT4 or FT3) at four time points during the first 12 months after delivery. For details on the 186 women representing the population assayed for gestational screening in strategies [h] through [k], see the Summary section of Results*.

*Progression to PH among the PPT cases detected were 34/63 (54% [strategy a]), 16/26 (61.5% [strategy b]), 16/25 (64% [strategy c]), 23/39 (59% [strategy d]), 24/42 (57.1% [strategy e]), 28/48 (58.3% [strategy f]), 29/54 (53.7% [strategy g]), 22/40 (55% [strategy h]), 22/42 (52.4% [strategy i]), 25/46 (54.3% [strategy j]), 25/49 (51% [strategy k]). The most favorable performance for each parameter (sensitivity through cost) is typed boldface. Diagnostic accuracy above 90% is highlighted by the gray background*.

Starting from strategy [d], there are costs to pay, which vary depending on the type of gestational screening (universal or selective), the thyroid Ab measured (TPOAb alone or both TPOAb and TgAb), and threshold of positivity for thyroid Ab. With the first four strategies ([d] through [g]), history taking and questionnaire of fish consumption are disregarded. Of these four strategies, the most rewarding is strategy [g] (assay of TPOAb and TgAb, either one considered positive when above 60 U/ml with our kits), since it allows to detect 86% of all PPT cases in the whole cohort with a mere 3% false positive rate, and at cost of Euro 290 per each case of PPT detected. This cost is 82% greater than that of strategy [d] (assay of TPOAb alone, considered positive when above the upper normal limit of 100 U/ml) in absolute terms, and 32% greater in relative terms (cost per each PPT detected). However, these greater costs are counterbalanced by a remarkable 24% increase in sensitivity and 2.4-fold lower false negative rate of strategy [g] vs. strategy [d].

Strategies [h] through [k] are the corresponding counterparts of strategies [d] through [g], the difference being that history taking and fish consumption are taken into consideration to render gestational screening selective as opposed to universal. Upon comparing *vis-à-vis* each pair of strategies ([h] vs. [d], [i] vs. [e], [j] vs. [f], and [k] vs. [g]), the selective one is consistently associated with greater specificity, lower false positive rate, comparable false negative rate, overall diagnostic accuracy >92%, lower absolute costs (gestational, postpartum, and total), and lower costs per each case of PPT or PH detected (Table 3).

## Discussion

### Principal Findings

Here we show that the costless screening of pregnant women for risk of developing PPT by simply inquiring on the presence/absence of three items results in categorization of women with different risks. These items are (i) AINTD in the woman herself, (ii) AINTD in at least one consanguineous relative, (iii) thyroid disease in at least one consanguineous relative. At one extreme, women negative for all three histories (a condition that we termed category I) or women positive only for personal history of AINTD (a condition that we termed category V) have no more than 10% risk of developing PPT. At the other extreme, women positive for all three histories (category II) or women positive for both personal and familial history of AINTD (category IV) have from 80 to 100% risk of developing PPT. The remaining four categories have intermediate risks between the two extremes.

We also show that the additional inquiry on seafood consumption, so as to stratify women in groups A through E, refines the risk. For instance, in category I and category V women, the overall corresponding risk for PPT of 9.7 and 3.8% doubles in group A women (22.2 and 8.0%, respectively). As a further instance, the small oily fish consumers (group B) have no risk at all if they belong to category I, and a 33% risk if they belong to category IV.

### Results and Clinical Implications

As a practical consequence of stratifications of pregnant women based on the costless inquiries on both the three histories and the fish consumption, one can omit the gestational biochemical screening for PPT risk in a good number of women, and can also omit the postpartum periodic biochemical assessment of thyroid function in a fraction of those women. By doing so, a few women with PPT will be lost. Finally, we have quantitated the statistical performance of different strategies and associated costs (gestational biochemical screening, if done, plus repeat postpartum monitoring). The following costs are given assuming of cohort of 1,000 women, of whom 153 develop PPT, based on our PPT rate of 15.3%. At one extreme, omitting universal gestational screening but performing universal postpartum screening of thyroid function is, of course, the most rewarding strategy (which we have called [a]) in terms of diagnosing all cases of PPT. However, it is also the most expensive. Total costs will be Euro 80,000 or Euro 523 per each case of PPT diagnosed. At the other extreme, the far cheapest strategy (total costs of Euro 5,040 or Euro 83 per each case of PPT diagnosed) is strategy [c], which is based on the costless inquiries on both the three histories and the fish consumption and selected postpartum monitoring of only 6.3% women in the cohort, namely all category II plus all category IV women except group B, plus group A women in category III. However, this strategy misses 60% of women who will develop PPT. The classic strategy (which we have called [d]) of the universal gestational screening by assaying TPOAb, and subsequent postpartum monitoring of thyroid function in the TPOAb +ve women (the threshold of positivity being TPOAb levels above the upper normal limit), results in total costs of almost Euro 21,000 or Euro 220 per each PPT diagnosed.

As reported previously, (2) and now corroborated by data in Table 3, statistically more rewarding than strategy [d] is strategy [g], in which both TPOAb and TgAb are measured for gestational screening and the woman is considered at risk (and therefore selected for postpartum monitoring of thyroid function) when levels of either Ab are even in the upper part of the reference range (>60 U/ml, with a reference range of 0–100 U/ml). Indeed, compared to strategy [d], in strategy [g] sensitivity increases from 62 to 86% (meaning that the rate of cases of PPT missed decreases from 38 to 14%), and overall diagnostic accuracy changes insignificantly (90 and 88%, respectively), at a total cost 1.8-fold higher in absolute terms (Euro 38,000) but 1.3-fold higher in terms of per case of PPT caught (Euro 290). The 86% sensitivity of strategy [g] ranks second after the obvious 100% rate of strategy [a]. The advantages of a greater predictive value of measuring during gestation both TPOAb and TgAb, as opposed to TPOAb only, and of considering positive also levels in the upper normal range can be combined with the costless inquiries on both the three histories and the fish consumption so as to render the gestational screening selective [see strategy [k] in Table 3], rather than universal (see strategy [g] in Table 3). Compared to strategy [g], strategy [k] is associated with lower sensitivity (78 vs. 86%), but greater overall diagnostic accuracy (93 vs. 88%), due to the much greater positive predictive value (75 vs. 58%). Compared to strategy [g], strategy [k] is cheaper (total costs of Euro 21,660 vs. 38,100, or 182 vs. 290 per each PPT caught).

### Strengths and Limitations

We think there are strenghts in our work. First, to the best of our knowledge, it is unprecedented. Second, depending on the particular scope of given studies, one may aim to maximize the number of women who, after delivery, will develop PPT or, in contrast, one may aim to minimize such number. Especially if faced with lack of fundings, investigators may resort to the costless inquiries on both the three histories and the fish consumption to stratify the cohort of women in those at high risk of PPT or those at low risk. Third, based on the available budget, one can estimate which strategy fits best the budget knowing the approximate yield of that strategy. One limitation is that our costs cannot be applied worldwide, not only because of intrinsic differences in the costs of kits, but also because the underlying epidemiology of PPT varies greatly (up to 20-fold) from one geographical area to another [see Introduction], ours being an area of mild iodine deficiency (10). A second limitation is that the distribution of the three-history categories and of the fish consumption groups may vary from one area to another. A further limitation is that the weight of presence/absence of each of the three histories and consumption of meat or certain fish as risky or protective factors for PPT development may vary on a geographical basis.

### Conclusions

The inclusion of histories to thyroid antibody status could improve antenatal screening of PPT. Identification of subgroups of pregnant women having different risks for PPT is feasible with the costless evaluation of clinical history for autoimmune diseases and seafood consumption, an evaluation that drives gestational screening and postpartum monitoring. Minimal loss of sensitivity, specificity and diagnostic accuracy, compared with universal postpartum monitoring, will be offset by significant savings.

## Data Availability Statement

The raw data supporting the conclusions of this article will be made available by the authors, without undue reservation, to any qualified researcher.

## Ethics Statement

The studies involving human participants were reviewed and approved by the Regional Department of Health, Programma per lo Sviluppo del Servizio Santiario Regionale, prot. no. 3/Dip/2007.

## Author Contributions

SB designed the study, collected, and analyzed data and wrote the manuscript.

## Conflict of Interest

The author declares that the research was conducted in the absence of any commercial or financial relationships that could be construed as a potential conflict of interest.
